# Evaluation of Two Formulations of Chlorantraniliprole as Maize Protectants for the Management of *Prostephanus truncatus* (Horn) (Coleoptera: Bostrychidae)

**DOI:** 10.3390/insects12030194

**Published:** 2021-02-25

**Authors:** Maria C. Boukouvala, Nickolas G. Kavallieratos

**Affiliations:** Laboratory of Agricultural Zoology and Entomology, Department of Crop Science, Agricultural University of Athens, 75 Iera Odos Str., 11855 Athens, Attica, Greece; mbouk@aua.gr

**Keywords:** larger grain borer, anthranilic diamide, formulations, stored maize, grain protectants

## Abstract

**Simple Summary:**

*Prostephanus truncatus* (Horn) (Coleoptera: Bostrychidae) is a major insect pest of stored maize and dried tubers of cassava, but also a wood-boring species. In the current study, we evaluated two chlorantraniliprole formulations, WG (wettable granule) and SC (suspension concentrate), as maize protectants against *P. truncatus* adults at 20, 25 and 30 °C. Both formulations performed similarly. The highest mortality was noted in chlorantraniliprole WG, at 10 ppm and 30 °C (98.9%), followed by chlorantraniliprole SC (96.1%), at the same dose and temperature. WG formulation was more effective at 10 ppm and 25 °C (92.8%) than SC formulation (89.4%). No progeny production was noted on maize treated the WG formulation at 20 and 30 °C. The SC formulation caused complete offspring suppression at 10 ppm at all three tested temperatures. The results indicate that chlorantraniliprole is an effective compound with a high insecticidal activity against *T. truncatus* that depends on temperature, dose and exposure.

**Abstract:**

The larger grain borer, *Prostephanus truncatus* (Horn) (Coleoptera: Bostrychidae) is one of the most destructive insect pests of stored maize and dried tubers of cassava, and a wood-boring species. In the present study, we examined two chlorantraniliprole formulations, WG (wettable granule) with 350 g/kg active ingredient (a.i.) and SC (suspension concentrate) with 200 g/L a.i., as maize protectants against *P. truncatus* adults. Chlorantraniliprole formulations were applied as solutions at 0.01, 0.1, 1 and 10 ppm, and tested at 20, 25 and 30 °C. Both formulations performed similarly. After 7 days of exposure, the overall mortality provided by both formulations was very low (<17%). Seven days later, mortality was remarkably increased on maize treated with 1 and 10 ppm at 25 and 30 °C for both formulations. The highest mortality was noted in chlorantraniliprole WG, at 10 ppm and 30 °C (98.9%), followed by chlorantraniliprole SC (96.1%), at the same dose and temperature. WG formulation was more effective at 10 ppm and 25 °C (92.8%) than SC formulation (89.4%). No progeny production was noted on maize treated with the WG formulation at 20 and 30 °C. The SC formulation caused complete offspring suppression at 10 ppm at all three tested temperatures. The results of the present work indicate that chlorantraniliprole is an effective compound with a high insecticidal activity against *T. truncatus* on stored maize that depends on temperature, dose and exposure interval. The fact that chlorantraniliprole is a broad-spectrum insecticide, exhibiting low toxicity to mammals and beneficial arthropods, could be a valuable management tool in storage facilities.

## 1. Introduction

The novel insecticide, chlorantraniliprole belongs to the chemical class of anthranilic diamides [1,2] that exhibits low toxicity to mammals [3] or to beneficial arthropods [4,5] and high toxicity to insect targets [6]. This insecticide has a unique mode of action, i.e., it activates the ryanodine receptor in insects’ muscles causing the release of cellular calcium that provokes termination of feeding, lethargy, paralysis of muscles and eventually leads to death [2,3,7]. Its insecticidal activity has been proved very effective against pests belonging to Coleoptera [8,9], Diptera [10], Hemiptera [11], Isoptera [12], Lepidoptera [13,14,15] and Thysanoptera [16]. However, an extensive examination in the international bibliography revealed few data about the insecticidal activity of chlorantraniliprole against insect pests of stored products. For example, Saglam et al. [17] examined this compound as surface treatment on concrete, against different life stages (i.e., egg, young and old larvae, pupae and adults) of the confused flour beetle, *Tribolium confusum* Jacquelin du Val (Coleoptera: Tenebrionidae). Kavallieratos et al. [18] reported that two chlorantraniliprole formulations were effective against the Mediterranean flour moth, *Ephestia kuehniella* Zeller (Lepidoptera: Pyralidae), the psocid *Liposcelis bostrychophila* Badonnel (Psocoptera: Liposcelididae), the lesser grain borer, *Rhyzopertha dominica* (F.) (Coleoptera: Bostrychidae), the rice weevil, *Sitophilus oryzae* (L.) (Coleoptera: Curculionidae) and *T. confusum* on six different commodities (i.e., barley, maize, oats, peeled rice, whole rice and wheat).

The larger grain borer, *Prostephanus truncatus* (Horn) (Coleoptera: Bostrychidae) is one of the most destructive insect pests of stored maize and dried tubers of cassava [19,20,21,22,23]. *Prostephanus truncatus* is also a dangerous wood-boring species to several forest plants [23,24,25]. It is native to Central and South America [26] from where it was accidentally introduced to Africa, and first recorded in Tanzania in the late 1970s [27,28,29]. Afterwards, *P. truncatus* spread in many African countries has been very rapid [23,30,31,32] as it has been favored by the fact it was able to remain in forest habitats for short periods [33]. This dangerous species exhibits high potential to become further spread in other continents [23]. *Prostephanus truncatus* may survive marginally on triticale, rice and whole oat flakes, and on different types of flour, i.e., whole barley flour, white soft wheat flour, whole soft wheat flour, white hard wheat flour and whole rye flour, indicating its undetected dispersion in the warehouses [34]. On the other hand, maize infestation by *P. truncatus* can occur before harvest [35], when its presence is not easy to detected, and therefore it can be easily introduced in the storage facilities with the infested seeds [35,36].

*Prostephanus truncatus* has developed resistance to several insecticidal compounds used for the protection of cereals during storage, such as organophosphorus compounds and pyrethroids [23,37]. It is also tolerant to various diatomaceous earths (DEs) used as maize protectants [38,39]. Therefore, it is vital to examine new and environmentally friendly substances, with a broad-spectrum insecticidal activity for the control of this species, taking into account that abiotic conditions are crucial for its development and damage potential [40]. Thus, the objective of the present study is to evaluate the insecticidal efficacy of two commercial formulations of chlorantraniliprole on stored maize, against *P. truncatus* under different temperatures: 20, 25 and 30 °C. Progeny production of *P. truncatus* has been estimated as well.

## 2. Materials and Methods

### 2.1. Insect, Commodity and Insecticidal Formulations

*Prostephanus truncatus* was cultured on whole maize seeds at 30 °C, 65% relative humidity and continuous darkness. The adults used in the experiments were unsexed and <2 weeks old. All individuals were taken from a colony maintained since 2014 in the Laboratory of Agricultural Zoology and Entomology, Agricultural University of Athens. Clean and free of infestation and pesticides maize, *Zea mays* L. (var. Dias) was used in the experiments. The moisture content of maize was 11.6% as determined by a moisture meter (mini GAC plus, Dickey-John Europe S.A.S., Colombes, France). The following two chlorantraniliprole formulations were used for the experiments: Altacor^®^ WG (wettable granule) with 350 g/kg active ingredient (a.i.) and Coragen^®^ SC (suspension concentrate) with 200 g/L a.i., both provided by Dupont (Halandri, Greece).

### 2.2. Bioassays

The chlorantraniliprole formulations were applied as solutions at 0.01, 0.1, 1 and 10 ppm a.i. based on Kavallieratos et al. [18]. One kg lots of maize were laid out on different trays and sprayed with 3 mL of an aqueous solution that contained the appropriate volume of WG or SC. For that purpose, an AG-4 airbrush (Mecafer S.A., Valence, France) was used. The airbrush was cleaned with acetone after the application of each dose of each formulation. The treated lots were then put into 5 L glass jars and were manually shaken for 10 min in order to achieve equal distribution of the insecticide in the entire maize mass [18]. Two additional lots of 1 kg maize each were treated with 3 mL of distilled water, using a different AG-4 airbrush, and served as controls. Three samples of 20 g each were taken from each treated or untreated lot and put into small glass vials (7.5 cm diameter by 12.5 cm height) with a different scoop that was inside each jar. Samples were weighed with a Precisa XB3200D compact balance (Alpha Analytical Instruments, Gerakas, Greece) using a different thin layer for each weighing. The lids of the vials had a 1.5 cm diameter hole in the middle, which was covered by gauze to allow sufficient aeration inside the vial. Then, 20 adults of *P. truncatus* were separately put inside each vial. The upper internal part of each vial was coated by polytetrafluoroethylene (60 wt % dispersion in water) (Northern Products Inc., Woonsocket, RI, USA) to prevent insect escape. Subsequently, all vials were placed inside an incubator set at 20 °C and 65% relative humidity. After 7 and 14 days of exposure, mortality was determined under an Olympus stereomicroscope (Olympus SZX9; Bacacos S.A., Athens, Greece) by prodding each insect gently with a brush (Cotman 111 No. 000, Winsor and Newton, London, UK) to detect any movement. Different brushes were used per dose/formulation and controls. At the 14 day evaluation of mortality, all alive or dead parental individuals were discarded and the vials were placed again inside the incubator for an additional period of 45 days. Then, the vials were opened and the progeny production (dead or alive) was estimated as described. The experiment was replicated three times by preparing a new series of insects, vials and maize lots each time. The entire procedure was repeated for 25 and 30 °C at 65% relative humidity as aforementioned. Each bioassay for each temperature was prepared within a three-day period.

### 2.3. Data Analysis

Mortality was very low in the control vials (<5%), therefore any correction was not necessary for the mortality counts. Prior to analysis, the mortality data were arcsine square root-transformed to normalize variance [41,42]. Statistical analysis was carried out according to the repeated-measures model [43]. The repeated factor was the exposure interval and mortality was the response variable. Formulation, temperature and dose were the main effects. The associated interactions of the main effects were incorporated in the analysis. Progeny production counts were subjected to a three-way ANOVA. Formulation, temperature and dose were the main effects. Number of progeny was the response variable. The associated interactions of the main effects and progeny production in the control vials were considered into the analysis. Means were separated by the Tukey–Kramer honestly significant difference (HSD) test at 0.05 level of significance [44]. JMP 14 software [45] was used to perform all analyses.

## 3. Results

### 3.1. Mortality of P. truncatus Adults

Between exposure intervals, all main effects and the associated interactions formulation × dose and temperature × dose were significant (Table 1). Within exposure intervals, all main effects and the associated interaction exposure × temperature × dose were significant.

After 7 days of exposure, the overall mortality provided by both formulations was very low. At 30 °C, mortality was slightly higher than at 25 or 20 °C, reaching 14.4 and 16.7% for chlorantraniliprole WG and SC, respectively (Table 2 and Table 3). Seven days later, mortality was remarkably increased on maize treated with 1 and 10 ppm at 25 and 30 °C for both formulations. At 20 °C, moderate mortality was recorded at the same doses, not exceeding 40.0 and 45.6% for chlorantraniliprole WG (Table 2), and 36.7 and 46.1% for chlorantraniliprole SC (Table 3). The highest mortality was noted on maize treated with chlorantraniliprole WG at 10 ppm and 30 °C (98.9%), followed by chlorantraniliprole SC (96.1%), at the same dose and temperature. Furthermore, WG formulation was also more effective (92.8%) than SC formulation (89.4%) at 10 ppm and 25 °C.

### 3.2. Progeny Production of P. truncatus Adults

The main effects, temperature and dose, and the associated interaction temperature × dose, were significant (Table 4).

Concerning the controls for both formulations, the highest progeny production was recorded at 30 °C, followed by 25 °C and 20 °C (Table 5 and Table 6). The increase of dose reduced the progeny emergence for both formulations. The highest progeny production was noted at 0.01 and 0.1 ppm for both formulations. No progeny production was noted at 20 °C on maize treated with 1 ppm of chlorantraniliprole WG. The WG formulation suppressed completely the offspring emergence on maize treated with 10 ppm at 20 and 30 °C, while 0.2 adults per vial were found at 25 °C (Table 5). Regarding SC formulation, complete offspring suppression was achieved only at 10 ppm and all three tested temperatures (Table 6).

## 4. Discussion

Due to the high importance of *P. truncatus* and the elevated risk of its further expansion, previous research efforts have been conducted for the effective management of this species. For instance, an insecticidal formulation of spinosad, which is based on the secondary metabolites spinosyns A and D of the bacterium *Saccharopolyspora spinosa* Mertz and Yao (Actinomycetales: Pseudonocardiaceae) [46,47] at 0.5 ppm, was able to kill >90% of *P. truncatus* adults after 7 days on maize, at 25 or 30 °C and 55 or 75% relative humidity [48]. The pyrrole chlorfenapyr has also been proved very effective after 7 days of exposure against *P. truncatus* by causing 100% mortality on maize treated with 5 ppm at 30 °C and 55 or 75% relative humidity [49]. Similarly, 0.5 ppm of the pyrethroid deltamethrin killed 97.8% of the exposed adults, at 20 °C, while complete mortality (100%) was recorded at 25 and 30 °C, at the same exposure interval [50]. According to our findings, both chlorantraniliprole formulations became highly toxic against *P. truncatus* at 10 ppm after 14 days of exposure at 25 and 30 °C or at 1 ppm at 30 °C by killing >90% of the exposed adults, indicating that chlorantraniliprole may cause complete mortality to this species favorably at longer exposure intervals (>14 days). The enhanced diatomaceous earths (DEs), or the mixture of a DE with another active ingredient could be used as alternative compounds for the protection of stored maize against *P. truncatus*. For instance, the enhanced DE, DEA-P which is a mixture of abamectin and freshwater DE, caused almost complete mortality in all tested combinations of temperature and relative humidity at 150 ppm after 7 days of exposure [39]. More recently, Kavallieratos et al. [51] reported the high insecticidal efficacy (≥98%), at low doses (75 and 150 ppm) of DEA-P, on five different maize hybrids against *P. truncatus* after 7 days of exposure. Similar results were noted by Kavallieratos et al. [52] for *S. oryzae* and *R. dominica*. Furthermore, the combination of the DE Protect-It with spinosad or the pyrethroid deltamethrin at low doses, caused high mortalities (84.5–99.5%) on treated maize against *P. truncatus* adults, even after 7 days of exposure [53]. Consequently, the combination of a chlorantraniliprole formulation with DEs at low doses may be more effective than the separate application of each formulation for the control of *P. truncatus*, even in shorter exposure intervals (<14 days), an issue that merits further experimentation.

In the present study, both chlorantraniliprole formulations have been very effective and performed almost equally. This similar trend was also recorded by Kavallieratos et al. [18] for *E. kuehniella* larvae, *L. bostrychophila* adults, *R. dominica* adults, *S. oryzae* adults and *T. confusum* larvae. However, the efficacy of each formulation among species was influenced by the type of the commodity, dose and exposure. In this study, we found that temperature played an important role in their insecticidal efficacy. According to our results, the increase of temperature from 20 to 30 °C resulted in significantly higher mortalities for both formulations in the majority of the tested combinations. Temperature is a crucial factor on the effectiveness of several insecticides that are used as grain protectants against stored-product insects [54,55]. For example, Kavallieratos and Boukouvala [56] reported higher mortality levels to the khapra beetle, *Trogoderma granarium* Everts (Coleoptera: Dermestidae), at 35 °C than at 30 °C on concrete treated with a mixture of acetamiprid plus d-tetramethrin plus piperonyl butoxide. In a recent study, Kavallieratos et al. [57] found that the insecticidal activity of pirimiphos-methyl against the yellow mealworm, *Tenebrio molitor* L. (Coleoptera: Tenebrionidae), was influenced by temperature. Concretely, the authors recorded the highest mortality rates of this species at 35 °C, followed by 30, 25 and 20 °C. The high effectiveness of insecticides at elevated temperatures can be explained by the fact that insects, due to their high mobility, come into contact with treated grains more often [52]. In addition, increasing the significant metabolic activities of insects when exposed to high temperatures may render them more vulnerable to toxic agents [58]. However, in the case of *P. truncatus*, there is no clear pattern about the influence of temperature on the activity of various a.i. applied on maize. Based on previous studies, chlorfenapyr, chlorpyriphos-methyl, deltamethrin, fipronil, pirimiphos-methyl, spinosad and spinetoram performed variably among 20, 25 and 30 °C depending on the combinations of dose, relative humidity and exposure interval [48,49,50,58,59]. Our results indicate that in the vast majority of the tested combinations, mortality increased with the rise in temperature regardless of the chlorantraniliprole formulation. This may be happening because chlorantraniliprole belongs to a different class of insecticides than the aforementioned a.i. [60,61,62,63,64,65,66,67,68,69,70]. For example, pyrethroids perform differently than organophosphates in changes of temperature given that, generally, the former are negatively while the latter are positively associated with the increase of temperature [57,71,72]. Further studies are needed to clarify this issue.

In our study, the progeny production of *P. truncatus* was very low for both formulations at 10 ppm. No offspring were noted on maize treated with chlorantraniliprole SC at any temperature, while in the case of chlorantraniliprole WG, 0.2 adults per vial were found at 25 °C. This is an important finding since chlorantraniliprole suppressed both the exposed adults and progeny production regardless of temperature. The suppression of progeny production at 25 and 30 °C is associated with the high adult mortality 14 days post-exposure. However, previous studies have shown that offspring emergence was not totally avoided even when the mortality was 100% or close to 100% on maize treated with chlorfenapyr [49] or pirimiphos-methyl and spinosad [50] or spinosad and spinetoram [58]. Most likely, females oviposited before dying, therefore some progeny were produced that eventually died [50]. The lethargy and paralysis that chlorantraniliprole induced to the exposed adults [1,2] may have not allowed them to lay eggs before their death, an issue that could further explain the absence of offspring in our experiments. This could also be the case for 20 °C since parental mortality ranged between 45.6 and 46.1% for both chlorantraniliprole formulations at 10 ppm. Additionally, 20 °C does not favor the activity of *P. truncatus* [73], thus its development becomes slow at this temperature level.

## 5. Conclusions

To conclude, the present study indicates that chlorantraniliprole is a promising a.i. for the management of *P. truncatus* because it provides high insecticidal activity to parental adults but also suppresses progeny production on stored-maize. However, temperature, exposure and dose should be taken into account if control measures against *P. truncatus* include chlorantraniliprole, regardless of WG or SC formulations. Since chlorantraniliprole exhibits activity against several other stored-product pests [18] and presents a friendly toxicological profile [3,4,5], it could be considered as an additional tool for the protection of stored-grain commodities.

## Figures and Tables

**Table 1:** Multivariate Analysis of Variance (MANOVA) parameters for main effects and associated interactions for mortality levels of *Prostephanus truncatus* adults between and within exposure intervals (total degrees of freedom, DF = 192).

Between Exposure Intervals			
Source	DF	*F*	*p*
Formulation	1	6.4	0.01
Temperature	2	131.1	<0.01
Dose	3	232.3	<0.01
Formulation × temperature	2	1.6	0.21
Formulation × dose	3	5.3	0.01
Temperature × dose	6	11.4	<0.01
Formulation × temperature × dose	6	1.4	0.21
**Within Exposure Intervals**			
Source	DF	*F*	*p*
Exposure × formulation	1	9.8	0.01
Exposure × temperature	2	43.7	<0.01
Exposure × dose	3	187.7	<0.01
Exposure × formulation × temperature	2	1.6	0.21
Exposure × formulation × dose	3	2.1	0.10
Exposure × temperature × dose	6	7.4	<0.01
Exposure × formulation × temperature × dose	6	0.5	0.91

**Table 2:** Mean mortality (% ± SE) of *Prostephanus truncatus* adults after 7 and 14 days on maize treated with the WG formulation of chlorantraniliprole, at four doses (0.01, 0.1, 1 and 10 ppm) under three temperatures (20, 25 and 30 °C). Within each column, means followed by the same lowercase letter are not significantly different (in all cases DF = 3, 35, Tukey–Kramer HSD test at 0.05). Within each row, means followed by the same uppercase letter are not significantly different (in all cases DF = 2, 26, Tukey–Kramer HSD test at 0.05). Where no letters exist, no significant differences were recorded.

	Exposure: 7 Days				
Temperature	20 °C	25 °C	30 °C		
Dose (ppm)				*F*	*p*
0.01	1.1 ± 0.7 AB	0.0 ± 0.0 Bb	6.1 ± 2.5 Ab	5.3	0.01
0.1	2.2 ± 0.9 B	4.4 ± 1.6 Bab	10.6 ± 2.7 Aab	6.6	0.01
1	2.7 ± 1.2 B	6.1 ± 2.5 ABab	11.7 ± 2.2 Aab	5.5	0.01
10	3.9 ± 1.4 B	9.4 ± 2.9 ABa	14.4 ± 1.6 Aa	6.4	0.01
*F*	0.9	4.7	4.1		
*p*	0.45	0.01	0.01		
	**Exposure: 14 Days**				
				*F*	*p*
0.01	7.2 ± 1.7 b	11.1 ± 4.5 d	25.0 ± 9.5 b	2.6	0.10
0.1	12.8 ± 1.2 Bb	34.4 ± 5.2 Ac	49.4 ± 7.4 Ab	13.4	0.01
1	40.0 ± 3.6 Ca	78.9 ± 3.8 Bb	92.2 ± 2.1 Aa	51.9	<0.01
10	45.6 ± 2.9 Ba	92.8 ± 2.8 Aa	98.9 ± 2.3 Aa	64.7	<0.01
*F*	41.0	59.1	33.0		
*p*	<0.01	<0.01	<0.01		

**Table 3:** Mean mortality (% ± SE) of *Prostephanus truncatus* adults after 7 and 14 days on maize treated with the SC formulation of chlorantraniliprole, at four doses (0.01, 0.1, 1 and 10 ppm) under three temperatures (20, 25 and 30 °C). Within each column, means followed by the same lowercase letter are not significantly different (in all cases DF = 3, 35, Tukey–Kramer HSD test at 0.05). Within each row, means followed by the same uppercase letter are not significantly different (in all cases DF = 2, 26, Tukey–Kramer HSD test at 0.05). Where no letters exist, no significant differences were recorded.

	Exposure: 7 Days				
Temperature	20 °C	25 °C	30 °C		
Dose (ppm)				*F*	*p*
0.01	1.1 ± 0.7	1.7 ± 0.8 b	3.9 ± 1.1 b	2.4	0.11
0.1	2.2 ± 0.9	2.2 ± 0.9 ab	5.0 ± 1.2 b	2.0	0.16
1	2.8 ± 0.9 B	6.1 ± 1.6 Bab	15.6 ± 2.8 Aa	10.5	0.01
10	3.3 ± 1.2 B	7.2 ± 1.5 ABa	16.7 ± 3.2 Aa	9.6	0.01
*F*	1.0	4.6	9.0		
*p*	0.43	0.01	0.01		
	**Exposure: 14 Days**				
				*F*	*p*
0.01	7.8 ± 1.2 b	8.9 ± 1.1 b	12.8 ± 2.5 b	1.5	0.25
0.1	10.0 ± 1.4 b	11.1 ± 2.2 b	17.2 ± 2.2 b	2.8	0.08
1	36.7 ± 4.2 Ba	76.7 ± 5.0 Aa	90.6 ± 1.6 Aa	35.5	<0.01
10	46.1 ± 5.3 Ba	89.4 ± 3.5 Aa	96.1 ± 1.4 Aa	39.9	<0.01
*F*	32.5	78.9	207.7		
*p*	<0.01	<0.01	<0.01		

**Table 4:** ANOVA parameters for progeny production of *Prostephanus truncatus* (total DF = 269).

Source	DF	*F*	*p*
Formulation	1	0.3	0.59
Temperature	2	9.0	0.01
Dose	4	89.7	<0.01
Formulation × temperature	2	0.7	0.50
Formulation × dose	4	0.5	0.72
Temperature × dose	8	2.0	0.05
Formulation × temperature × dose	8	0.6	0.81

**Table 5:** Progeny production (adults per vial ± SE) of *Prostephanus truncatus* on maize treated with the WG formulation of chlorantraniliprole at five doses (0, 0.01, 0.1, 1 and 10 ppm) under three temperatures (20, 25 and 30 °C) 45 days after the removal of parental adults. Within each column, means followed by the same lowercase letter are not significantly different (in all cases DF = 4, 44, Tukey–Kramer HSD test at 0.05). Within each row, means followed by the same uppercase letter are not significantly different (in all cases DF = 2, 26, Tukey–Kramer HSD test at 0.05). Where no letters exist, no significant differences were recorded.

Temperature	20 °C	25 °C	30 °C		
Dose (ppm)				*F*	*p*
0	8.8 ± 2.2 a	12.4 ± 2.3 a	15.0 ± 1.5 a	3.2	0.06
0.01	7.1 ± 2.1 ab	7.8± 1.4 ab	11.2 ± 2.6 a	0.5	0.60
0.1	2.3 ± 1.3 Bbc	4.4 ± 1.5 ABbc	9.6 ± 1.8 Aa	4.6	0.02
1	0.0 ± 0.0 c	0.8 ± 0.5 cd	0.3 ± 0.2 b	1.2	0.31
10	0.0 ± 0.0 c	0.2 ± 0.2 d	0.0 ± 0.0 b	1.0	0.38
*F*	10.4	20.9	25.6		
*p*	<0.01	<0.01	<0.01		

**Table 6:** Progeny production (adults per vial ± SE) of *Prostephanus truncatus* on maize treated with the SC formulation of chlorantraniliprole at five doses (0, 0.01, 0.1, 1 and 10 ppm) under three temperatures (20, 25 and 30 °C) 45 days after the removal of parental adults. Within each column, means followed by the same lowercase letter are not significantly different (in all cases DF = 4, 44, Tukey–Kramer HSD test at 0.05). Within each row, means followed by the same uppercase letter are not significantly different (in all cases DF = 2, 26, Tukey–Kramer HSD test at 0.05). Where no letters exist, no significant differences were recorded. Where dashes exist, no analysis was performed.

Temperature	20 °C	25 °C	30 °C		
Dose (ppm)				*F*	*p*
0	8.7 ± 1.8 a	12.3 ± 3.6 a	14.8 ± 2.0 a	2.0	0.16
0.01	5.2 ± 1.5 a	6.9 ± 2.5 ab	13.2 ± 2.6 a	2.0	0.15
0.1	3.2 ± 0.7 ab	4.3 ± 0.9 ab	7.8 ± 2.0 a	1.0	0.38
1	1.8 ± 1.4 bc	2.4 ± 1.6 bc	0.4 ± 0.3 b	0.6	0.56
10	0.0 ± 0.0 c	0.0 ± 0.0 c	0.0 ± 0.0 b	-	-
*F*	13.7	6.8	23.8		
*p*	<0.01	0.01	<0.01		

## Data Availability

Data is contained within the article.
